# Predicting Vertebral Bone Strength Using Finite Element Analysis for Opportunistic Osteoporosis Screening in Routine Multidetector Computed Tomography Scans—A Feasibility Study

**DOI:** 10.3389/fendo.2020.526332

**Published:** 2021-01-19

**Authors:** Nithin Manohar Rayudu, Michael Dieckmeyer, Maximilian T. Löffler, Peter B. Noël, Jan S. Kirschke, Thomas Baum, Karupppasamy Subburaj

**Affiliations:** ^1^ Engineering Product Development (EPD) Pillar, Singapore University of Technology and Design (SUTD), Singapore, Singapore; ^2^ Department of Diagnostic and Interventional Neuroradiology, Klinikum rechts der Isar, Technische Universität München, Munich, Germany; ^3^ Department of Radiology, Perelman School of Medicine, University of Pennsylvania, Philadelphia, PA, United States

**Keywords:** multidetector computed tomography, spine, finite element analysis, osteoporosis, opportunistic screening

## Abstract

**Purpose:**

To investigate the feasibility of using routine clinical multidetector computed tomography (MDCT) scans for conducting finite element (FE) analysis to predict vertebral bone strength for opportunistic osteoporosis screening.

**Methods:**

Routine abdominal MDCT with and without intravenous contrast medium (IVCM) of seven subjects (five male; two female; mean age: 71.86 ± 7.40 years) without any bone disease were used. FE analysis was performed on individual vertebrae (T11, T12, L1, and L2) including the posterior elements to investigate the effect of IVCM and slice thickness (1 and 3 mm) on vertebral bone strength. Another subset of data from subjects with *vs*. without osteoporotic vertebral fractures (n = 9 age and gender-matched pairs) was analyzed for investigating the ability of FE-analysis to differentiate the two cohorts. Bland-Altman plots, box plots, and coefficient of correlation (R^2^) were calculated to determine the variations in FE-predicted failure loads for different conditions.

**Results:**

The FE-predicted failure loads obtained from routine MDCT scans were strongly correlated with those from without IVCM (R^2 =^ 0.91 for 1mm; R^2^ = 0.92 for 3mm slice thickness, respectively) and different slice thicknesses (R^2^ = 0.93 for 1mm *vs*. 3mm with IVCM). Furthermore, a good correlation was observed for 3mm slice thickness with IVCM *vs*. 1mm without IVCM (R^2^ = 0.87). Significant difference between FE-predicted failure loads of healthy and fractured patients was observed (4,705 ± 1,238 *vs*. 4,010 ± 1,297 N; p=0.026).

**Conclusion:**

Routine clinical MDCT scans could be reliably used for assessment of fracture risk based on FE analysis and may be beneficial for patients who are at increased risk for osteoporotic fractures.

## Introduction

Osteoporosis is a skeletal disorder that occurs due to bone loss and deterioration of bone microarchitecture (1, 2). However, these changes remain undetected until a fragility fracture happens and then it significantly affects the quality of life and is associated with increased morbidity and mortality (3–6). Thus, the assessment of bone health at an early stage of the disease is crucial in terms of treatment initiation and fracture prevention.

Currently, dual-energy X-ray absorptiometry (DXA) is considered as the gold standard for osteoporosis diagnosis (7, 8). Even though DXA-based aerial bone mineral density (aBMD) has high clinical relevance, its effectiveness in predicting fragility fractures is limited (8, 9). Studies have shown that subjects with normal aBMD values suffered from osteoporotic fractures and vice versa (8, 9). Quantitative computed tomography (QCT) can be used in place of DXA to measure volumetric BMD from the attenuation values using a calibration phantom (10–13). Considering the complex three-dimensional microstructure of bone, QCT imaging provides more information required in assessing bone quality than DXA (10, 14, 15). Three-dimensional patient-specific finite element (FE) models derived from medical images (realistic 3D anatomy, heterogeneous material properties mapping based on attenuation values, and loading and boundary conditions to predict response) have been increasingly used for solving biomechanical-related clinical problems, including bone strength predictions (16–19).

Multidetector computed tomography (MDCT) derived quantitative measures using advanced computational methods, including texture analysis and patient-specific FE analysis, are emerging to become clinically relevant metrics in identifying patients at the risk of having osteoporotic fractures. In the literature, most of the studies were performed in research settings, where MDCT images are acquired with high resolution and without intravenously applied contrast medium. However, in routine clinical settings, MDCT scans are frequently acquired with intravenous contrast medium (IVCM) and sagitally reformatted with a slice thickness of up to 3 mm to assess the fracture status at the spine (20, 21).

The purpose of the current study was to assess the feasibility of opportunistic osteoporosis screening by finite element analysis in routinely acquired MDCT scans. To achieve the purpose mentioned above, we set out to investigate the following objectives:

1. Compare the failure load predicted by the FE-model generated from 1 and 3mm image data without intravenous contrast medium to study the effect of slice thickness,
2. Compare the failure load predicted by the FE-model generated from 1 and 3mm image data with and without intravenous contrast medium to study the effect of IVCM, and
3. Compare the failure load predicted by the FE-model generated from 1mm without IVCM and 3mm image data with IVCM to explore the possibility of using routine clinical image data for opportunistic osteoporosis screening.
4. Compare the failure load predicted by the FE-model generated from subjects with osteoporotic vertebral fractures and gender-/age-matched controls to explore the feasibility of using FE analysis for differentiating these cohorts.

## Materials and Methods

**Figure 1:**
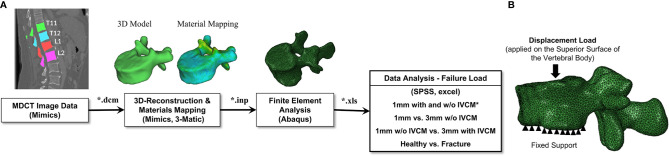
**(A)** Schematic representation of the data generation and analysis methodology followed in the study. The vertebrae were delineated on the images acquired to generate a 3D model of the geometry to be used in the downstream finite element analysis protocol to predict the bone strength. *IVCM means intravenous contrast medium used to acquire contrast enhanced MDCT images. **(B)** Loading and boundary conditions applied in performing the finite element analysis of the full vertebra (with posterior elements). Fixed support represents the zero displacement in all directions at the inferior surface of the vertebral body. Displacement load was applied on the superior surface of the vertebral body to predict the failure load.

**Figure 1A** shows the schematic representation of the methodology followed in generating and analyzing the data to study the objectives described in the introduction section. The proposed method involves four major sub-sections, namely MDCT data acquisition, 3D reconstruction of the anatomical models from the image data, finite element analysis including meshing, material properties mapping, and applying loading and boundary conditions, and data analysis.

### Subjects

This retrospective study was reviewed and approved by the local institutional review board. Due to retrospective nature, the ethics committee waived the requirement of written informed consent for participation.

For investigating the effects of posterior elements, slice thickness, and IVCM, a total of seven subjects (five males and two females, median age: 71.86 ± 7.40 years) who underwent routine abdominal MDCT were included in this study, as outlined previously (20). Subjects with known history of bone pathologies, including metastases, spine fractures, metabolic, or hematological disorders aside from osteoporosis were excluded. Patients who underwent routine non-contrast abdominal MDCT and immediately followed contrast-enhanced abdominal MDCT at our institution were retrospectively identified by a board-certified radiologist in our institution’s digital image archive (PACS).

To explore the feasibility of using finite element analysis for differentiating healthy from the fractured cohort, a group of subjects with osteoporotic vertebral fractures (n_f_ = 9; four males, five females, mean age: 75.44 ± 10.19 years) and gender-/age-matched healthy controls without vertebral fracture (n_h_ = 9; four males, five females; mean age: 71.44 ± 10.05 years) were included. Patients with osteoporotic vertebral fractures were retrospectively identified by a board-certified radiologist based on the available routine abdominal contrast enhanced MDCT scan data in our institution’s digital image archive (PACS). These patients had a history of cancer (such asesophageal, colorectal, or breast cancer). They underwent the MDCT examination as long-term follow-up to rule out tumor recurrence.

### Multidetector Computed Tomography Imaging

Subjects identified for investigating the effects of posterior elements, slice thickness, and IVCM underwent abdominal non-contrast-enhanced MDCT scans, immediately followed by contrast-enhanced MDCT scans at a 64-row MDCT scanner (Somatom Sensation Cardiac 64, Siemens Medical Solution, Erlangen, Germany). The scanning parameters were 120 kVp of tube voltage, 200 mAs of adapted tube load averaged, and 0.6 mm of collimation. Acquired data were sagitally reformatted and reconstructed with slice thicknesses of 1 and 3mm, since the spine image reformations with a sagittal slice thickness of 3mm are the standard in clinical routine at our hospital. Intravenous contrast medium (Iomeron 400, Bracco, Konstanz, Germany) was administered through high-pressure injector (Fresenius Pilot C, Fresenius Kabi, Bad Homburg, Germany). The intravenous contrast medium injection was carried out with a delay of 70 s, a flow rate of 3 ml/s, and a body weight-dependent dose (80 ml for bodyweight up to 80 kg, 90 ml for bodyweight up to 100 kg, and 100 ml for bodyweight over 100 kg). Segmentations of the vertebrae (T11 to L2; a total of n = 28 vertebrae) were performed by a radiologist using MITK (Medical Imaging Interaction Toolkit; www.mitk.org) software program for these sub-analyses.

MDCT scans for the subjects identified to explore the feasibility of using FE analysis to differentiate healthy from the fractured were acquired using a 256-row scanner (iCT, Philips Healthcare, Best, the Netherlands). The scanning parameters, as well as the protocol for administering intravenous contrast medium, are the same as mentioned above. Sagittal reformations of the spine were reconstructed with a slice thickness of 3 mm. Vertebrae for this sub-analysis (L1–L4; a total of n = 27 vertebrae in each cohort) were segmented, and the presence of vertebral fractures was determined and documented by a board-certified radiologist using the sagittal reformations of the spine.

### Finite Element Modeling

The acquired non-contrast-enhanced and contrast-enhanced MDCT images along with segmented 3D masks for vertebrae were imported to the medical image analysis software program, Mimics (Materialise NV, Leuven, Belgium), for downstream image analysis. The segmented mask data of each vertebra were converted into a 3D geometric model before importing into 3-Matic software program (Materialise NV, Leuven, Belgium) to generate finite element mesh using a linear tetrahedral element (C3D4 in Abaqus element library). Once the meshing was performed, material properties of the vertebra were derived based on the density (ρ)—HU and density (ρ)—elastic modulus (E) relationship, shown in **Table 1**, and then mapped onto the finite element mesh.

**Table 1:** Vertebral bone material mapping relations used in the current finite element study (22).

Property	Mapping relations
Apparent density (ρapp)	ρapp = 47 + 1.122 * HU HU—Hounsfield unit
Ash density (ρash)	ρash= 0.6 * ρapp
Elastic modulus (E)	Ez = -349 + 5.82 * ρapp Ex= Ey = 0.333 Ez Z-axial direction of the vertebra
Shear modulus (G)	Gxy = 0.121 Ez Gxz = Gyz = 0.157 Ez
Maximum principal stress limit (σ)	σ = 137 * ρash 1.88, ρash < 0.317 σ = 114 * ρash 1.72, ρash > 0.317
Plastic strain (ϵAB)	ϵAB = -0.00315 + 0.0728 ρash
Minimum principal stress limit (σmin)	σmin = 65.1 * ρash 1.93

Also, for producing mesh-independent solution, we have performed mesh sensitivity analysis by varying the maximum edge length from 1.0 to 3.0 mm with an interval of 0.5 mm (1.0-, 1.5-, 2.0-, 2.5-, and 3.0-mm sizes were considered). The analysis showed that 2 mm element edge length produced mesh-independent solution based on failure load convergence and the same was used in all the developed finite element models for further analysis.

### Failure and Displacement Load Analysis

The meshed and material mapped model was exported from the Mimics in the Abaqus input format (*.inp). This file was then imported to a commercial finite element analysis software, Abaqus ver. 6.10 (Hibbitt, Karlsson, and Sorensen, Inc., Pawtucket, RI, USA) and the loading and displacement boundary conditions for the 3D vertebra model were applied and the model is analyzed. In this study, the vertebra was subjected to compression load and simulated to obtain the failure load (16, 22). The inferior surface of the vertebral body was fully constrained in all the directions and then a displacement load was applied on the superior surface of the vertebral body, as shown in **Figure 1B**. Transversely isotropic properties were given to the vertebra and the failure load was calculated. The failure load was defined as the peak of the force-displacement curve and it was considered as the bone strength (16, 22).

### Statistical Analysis

Statistical data analyses were performed using Microsoft Excel, Version 16.27 (2019) (Microsoft Corporation, Seattle, WA, USA) and SPSS (SPSS Inc., Chicago, IL, USA). The distributions of failure load were plotted and examined. T-test was performed to evaluate the effect of including posterior elements in the FE analysis on the FE-predicted failure load values and also to check the differences between thoracic and lumbar vertebrae to observe within patient vertebral correlation. The root-mean-square coefficient of variation (RMSCV) was calculated to quantify the reproducibility of the analysis. Linear regression models and the coefficient of correlation (R^2^) were used for evaluating the variability in failure loads obtained from the FE models developed from the images acquired under different scanning parameters (1mm with IVCM *vs*. 1mm w/o IVCM and 3mm with IVCM *vs*. 3mm w/o IVCM) for assessing the effect of intravenous contrast medium on the FE-predicted failure load. To evaluate the effect of different slice thicknesses on the FE-predicted failure load, we calculated the coefficient of correlation (R^2^) for different scanning conditions (1mm with IVCM *vs*. 3mm with IVCM, 1mm w/o IVCM *vs*. 3mm w/o IVCM). To study the feasibility of conducting opportunistic analysis using the routine clinical data, we calculated the coefficient of correlation (R^2^) between 1mm w/o IVCM (research data) and 3mm with IVCM (routine clinical data). In addition, we generated Bland-Altman plots (23) to assess the spread of the FE-predicted failure load values. Finally, a t-test was performed to compare the means of FE-predicted failure loads for the two cohorts (healthy *vs*. fractured patients).

## Results

### Effect of Vertebral Posterior Elements on Finite Element-Predicted Failure Load

**Figure 2:**
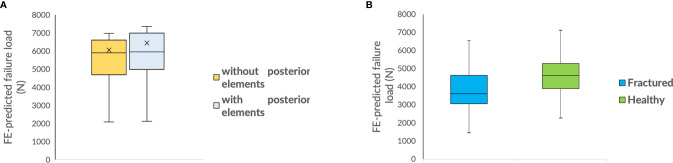
**(A)** Box plot comparing FE-predicted failure load of a vertebra with and without posterior elements; **(B)** Box plot comparing FE-predicted failure loads of healthy and fractured cohort.

Considering many of the FE studies reported in the literature only included the vertebral body for the strength prediction of the vertebra, we analyzed a sub-cohort of seven subjects T11 vertebra with and without posterior elements to study its effect on the FE-predicted failure load. The results showed a significant difference of 5.13 ± 3.05% (p<0.01) (**Figure 2A**), indicating the FE models of the vertebra with posterior elements have a higher failure load than the models without. Thus, for better accuracy and more realistic simulation, we have included the posterior elements for the modeling of the vertebrae in this study. We have repeated the simulations with and without posterior elements for reproducibility analysis and observed very low differences in the FE-predicted failure load values (RMSCV =2.72%, without posterior elements; RMSCV = 2.89%, with posterior elements). We have also observed significant difference in FE-predicted failure load values between with and without posterior elements 5.13 ± 3.05%, try 1; 5.53 ± 4.45%, try 2). We have also not observed much significant differences between thoracic and lumbar vertebrae (p=0.64). The mean FE-predicted failure load values for thoracic and lumbar vertebrae are shown in **Table 2**.

**Table 2:** Finite element (FE)-predicted vertebral failure load values (mean and std. dev) and level of significance for thoracic and lumbar vertebrae.

	Vertebral failure load	
	Thoracic vertebrae (T11 and T12)	Lumbar vertebrae (L1 and L2)
Mean value (N)	5,723.16	5,456.21
Std. dev (N)	2,989.82	2,191.53
p-value	0.64	

### Effect of Intravenous Contrast Medium on Finite Element-Predicted Failure Load

**Figure 3:**
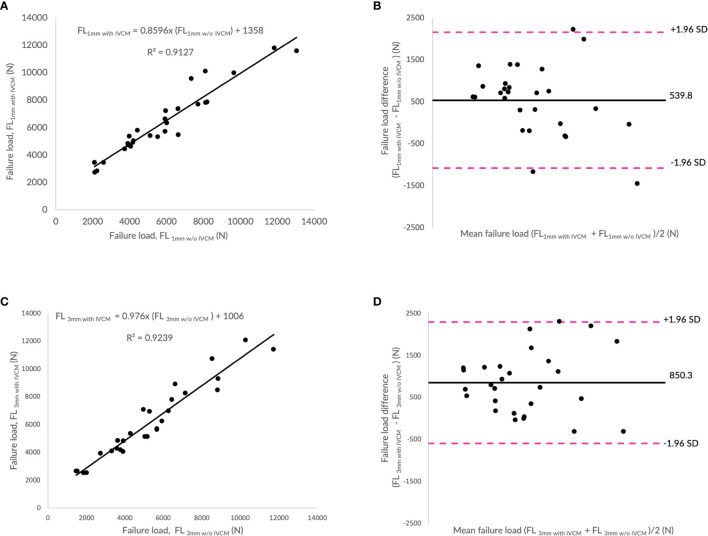
Effect of intravenous contrast medium (IVCM) on finite element (FE)-predicted failure load values. **(A)** Correlation plot between FE-predicted failure load values for 1mm with and without IVCM, **(C)** correlation plot between FE-predicted failure load values for 3mm with and without IVCM, and **(B, D)** Bland Altman plots representing the mean of FE-predicted failure load values versus difference between them in 1 and 3mm scan settings, respectively. Horizontal lines represent mean and dashed line ±1.96 standard deviation. FL represents FE-predicted vertebral failure load (N).

**Figures 3A, C** show the correlations between failure load values predicted by the FE analysis of the vertebrae modeled from the contrast-enhanced and non-contrast enhanced MDCT images at two different slice thicknesses (1mm w/o IVCM *vs*. 1mm with IVCM and 3mm w/o IVCM *vs*. 3mm with IVCM, respectively). Correlations between FE-predicted failure loads based on the images with and without IVCM were high at both, 1mm (R²=0.91) and 3mm slice thickness (R²=0.92). To assess the relationship between FE-predicted failure loads obtained from the models of data acquired with and without IVCM, we plotted the difference between failure loads of these two instances against the mean of them. **Figures 3B, D** show the Bland-Altman plots of FE-predicted failure loads obtained from images with and without IVCM with two different slices thicknesses, 1 and 3mm, respectively. A positive bias (540 N for 1mm and 850 N for 3mm) toward the data obtained with IVCM than without was evident in both plots (different slice thicknesses) and the data spread on both sides of the mean line appeared to be even in both plots.

### Effect of Slice Thicknesses on Finite Element-Predicted Failure Load

**Figure 4:**
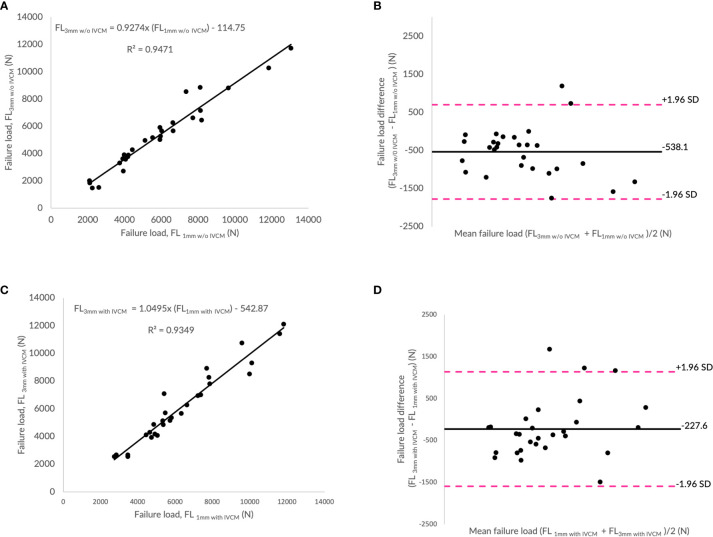
Effect of slice thickness on finite element (FE)-predicted failure load values. **(A)** Correlation plot between FE-predicted failure load values for 1 and 3mm without intravenous contrast medium (IVCM), **(C)** correlation plot between FE-predicted failure load values for 1 and 3mm with IVCM, and **(B, D)** Bland Altman plots representing the mean of FE-predicted failure load values versus difference between them in with and without IVCM scan settings, respectively. Horizontal lines represent mean and dashed line ±1.96 standard deviation. FL represents FE-predicted vertebral failure load (N).

**Figures 4A, C** show the correlations between failure load values predicted by the FE analysis of the vertebrae modeled from MDCT images with different slices thicknesses (1 and 3 mm) and with and without contrast medium (IVCM). FE-predicted failure loads with different slice thicknesses (1mm *vs*. 3mm w/o IVCM; 1mm *vs*. 3mm with IVCM) were found to be highly correlated in both instances of with (R²=0.93) and without contrast medium (R²=0.95). The interaction between slice thickness and contrast medium on the predicted failure loads was not significant (p>0.05). To assess the relationship between FE-predicted failure loads obtained from the models developed from the image data acquired at different slice thicknesses (1 and 3mm), we plotted the difference between failure loads of these two instances against the mean of them. **Figures 4B, D** show the Bland-Altman plots of FE-predicted failure loads obtained from images at two different slice thickness and with and without IVCM, respectively. A negative bias (−538 N for without IVCM and −228 N for with IVCM) toward the data obtained with 3mm than 1 mm was evident in both the plots (with and w/o IVCM) and the data spread on both sides of the mean line appeared to be even in both the plots.

### Feasibility of Using Routine Clinical Image Data for Finite Element Analysis

**Figure 5:**
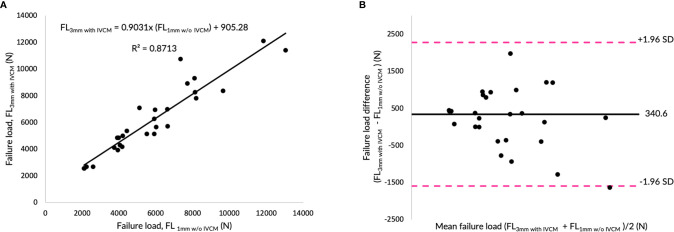
Routine clinical image data for finite element (FE)-Analysis. **(A)** Correlation plot between FE-predicted failure load values for high-resolution and routine image data, and **(B)** Bland Altman plot representing the mean of FE-predicted failure load values versus difference between them in for high-resolution and routine image data scan settings, respectively. Horizontal lines represent mean and dashed line ±1.96 standard deviation. FL represents FE-predicted vertebral failure load (N).

**Figure 5A** shows the correlations between failure load values predicted by the FE analysis of the vertebrae modeled from 1mm slice thickness w/o IVCM *vs*. 3mm slice thickness with IVCM. We found that the correlation between FE-predicted failure loads from routine clinical data and high-resolution data was high (R²=0.87). To assess the relationship between FE-predicted failure loads obtained from the models generated from these image data, we plotted the difference between failure loads of these two instances against the mean of them. **Figure 5B** shows the Bland-Altman plot of FE-predicted failure loads. A positive bias (341 N) toward the values obtained from the routine clinical data than high-resolution data was evident in the plot and the data spread on both sides of the mean line appeared to be even.

### Feasibility of Using Finite Element Analysis for Differentiating Healthy and Fractured Cohort

We analyzed a sub-cohort of nine subjects (with/without vertebral fractures) of depicted vertebrae (L1–L4) to explore the feasibility of using FE analysis of routine clinical MDCT images in differentiating healthy and fractured patients. The results showed a significant difference in FE-predicted failure load (4,705 ± 1,238 N for healthy and 4,010 ± 1,297 N for fractured patients; p=0.026) (**Figure 2B**), indicating the vertebrae in the healthy cohort have a higher failure load than in the fractured cohort.

## Discussion

In the current study, we evaluated the feasibility of using routine clinical MDCT data to generate finite element models for the opportunistic assessment of osteoporotic fracture risk based on FE-predicted vertebral strength. In routine clinical diagnostic image acquisition settings, MDCT image data is frequently reconstructed with a larger slice thickness and acquired with intravenous contrast medium administered. Our results suggest that the FE-predicted vertebral failure loads obtained from routine MDCT data, i) with contrast medium were slightly higher but strongly correlated with the values derived from the data without IVCM (R^2 =^ 0.91 for 1mm; R^2^ = 0.92 for 3mm) and ii) with a larger slice thickness were slightly lower but strongly correlated with the values derived from the data with a smaller slice thickness (R^2^ = 0.93 for 1mm *vs*. 3mm with IVCM). The routine clinical image data with IVCM also showed a high correlation with the high-resolution image data without IVCM (R^2^ = 0.87 for 3mm with IVCM *vs*. 1mm w/o IVCM). Furthermore, a considerable difference was observed between the FE-predicted failure loads of healthy and fractured cohort (4,705 ± 1,238 N *vs*. 4,010 ± 1,297 N; p=0.026). Thus, the routine clinical data could potentially be used for opportunistic assessment of osteoporotic fracture risk based on FE-predicted vertebral strength in patients who are at risk for osteoporotic fractures such as cancer patients (24).

In routine clinical settings, high slice thickness is used due to its advantages like reduced image noise (25). However, it suffers from partial volume effects. In this study, we observed that the increase in slice thickness from 1 to 3mm resulted in a slightly lower FE-predicted failure load, but with a strong correlation. This variation could be attributed to the process of generating the 3D geometric model of the anatomy from the segmented masks, which may have contributed to a difference in the overall volume of the model (26, 27). The stronger correlation with bias indicates only a shift in the predicted values while following the same trend. This result suggests that the FE-predicted failure load value for the models generated from the image data with a higher slice thickness possibly be lower than the one with thinner slice thickness and corrections may be needed to account for when using the value for diagnostic purpose or extrapolating it for other analyses.

FE-predicted failure loads obtained from the models generated from the image data acquired with and without contrast medium were strongly correlated. However, despite the stronger correlations between them, the values predicted from the data with IVCM were slightly higher at both instances with two different slice thicknesses (1 and 3mm). In routine clinical scans, intravenous contrast medium is administered before the CT scan to improve the image contrast and detection of pathological findings, thus improving the diagnostic accuracy. Studies have shown that the contrast medium absorbed by the vertebral body increases the signal intensity, which affects the material mapping step of the FE analysis workflow (28–30). In addition, the interplay of slice thickness (partial volume effect) and contract-enhancement (signal intensity) in the routine clinical MDCT images can have effect on the material properties assigned based on the image data and reconstructed geometric model of the anatomy for downstream finite element analysis to predict bone strength, which has not been studied extensively in the literature (10, 31). Thus, we can conclude from these results that there is a slight increase in the value predicted from the image data acquired with contrast medium than the one without independent of slice thickness while following the same trend. This finding is consistent with opportunistic BMD assessment in contrast-enhanced MDCT (32–34).

Opportunistic analysis of fracture risk using MDCT scans acquired for other purposes would reduce costs and radiation exposure. Moreover, it would allow conducting big retrospective clinical studies and analyses (20, 35, 36). Any abdominal, chest, or head and neck scan could be suitable for vertebral strength assessment. Recently, Schwaiger et al. demonstrated the feasibility of using retrospective positron emission tomography with computed tomography (PET/CT) data to opportunistically evaluate bone density and strength in men with prostate cancer (35). Mookiah et al. demonstrated the feasibility of using abdominal MDCT scans for evaluating bone quality using image textural parameters (20). These studies have demonstrated the feasibility that additional information that can be extracted from the scans acquired for other purposes, without the extra burden of radiation and scan time, to assist the clinician in making clinical decisions. In this study, we have evaluated the feasibility of using routinely acquired clinical image data for the diagnostic purpose (3mm slice thickness with contrast medium) to generate finite element model of the anatomy, map the material properties based on the image intensity, and simulate to predict the bone strength of the specific patient. Our results suggest that the FE-predicted failure load values from the routine clinical data are in very good correlation (R^2^ = 0.87) with the one predicted from the high-resolution image data. However, a slight positive bias toward the routine data was evident and the predicted failure load values are on the higher side than the high-resolution data. This result suggests that, with adjustment, the routine clinical data can potentially be used to conduct downstream finite element analysis.

Osteoporotic vertebral fractures are a significant determinant of the quality of life in the elderly, including increased back pain, impairment of mobility, and functional limitations on performing activities of daily living (37). Identifying subjects who are at the risk of having vertebral fractures is a crucial step in disease management and treatment. The effectiveness of dual-energy X-ray absorptiometry (DXA)-derived areal bone mineral density (aBMD), a standard osteoporosis diagnostic tool, in differentiating patients with fracture risk and monitoring treatment effect is limited (8, 9). Vertebral strength measures derived from validated FE modeling and analysis have shown potential in assessing fracture risk and detecting short-term treatment efficacy (16, 18, 22, 38, 39). In this study, we have explored the feasibility of using FE models generated from routine clinical image data in differentiating healthy from the fractured cohort. Our results suggest that there is a significant difference in the observed FE-predicted failure loads between healthy and fractured (4,705 ± 1,238 N *vs*. 4,010 ± 1,297 N; p=0.026). Besides, the predicted values are well within the range obtained from the research-level image data, as reported in the literature (16, 22). Thus, we can conclude from the results that the FE based measures could be used to assess fracture risk and differentiate the healthy and fractured cohorts.

The vertebral bone has two major load-bearing elements, i.e., vertebral body and posterior elements (facet joints). The majority of the reported studies have analyzed the vertebrae by modeling only the vertebral body due to difficulties and time associated with segmenting the posterior elements (22, 40, 41). However, advances in automated vertebrae segmentation algorithms, including artificial intelligence-driven ones, significantly reduced those issues (42–45). Considering approximately 10% of the load on the vertebral column transferred through the facet joints and posterior elements (46–48), we contend that it should be included in quantifying the strength of a vertebra. In this study, we have observed that the FE-predicted failure load values are higher when the posterior elements are included. Recent finite element studies (49–51) have shown improved accuracy in calculating vertebral bone strength through finite element analysis when the posterior elements are included in the analysis compared to not. Thus, for accurate calculation of failure load, the analysis should consider including the posterior elements in the model.

There are some associated limitations of the study which have to be taken into account when interpreting the results obtained. First, the segmentation of the vertebrae was performed manually, which was time-consuming. Automated segmentation algorithms have to be applied for widespread clinical use in the future. Second, this pilot study was carried out with a relatively small cohort size, which may have contributed to the higher variations observed in some of the analyses performed. Future studies have to evaluate the performance of opportunistic FE analysis in clinical routine MDCT data to predict incidental fractures in a longitudinal setting. Third, there were some outliers in observed failure load in all cases in both with/without contrast medium and with different slice thickness, which may have contributed to computing correlations and affected the comparison. Fourth, large differences between the FE-predicted failure load values were observed for a few subjects. This may be due to observed higher material stiffness in those, which may have resulted in a higher failure load under current loading configuration. Fifth, in this study, we have considered only static compression loading configuration for comparison purposes; however, in other loading configurations, the FE-predicted failure load values and the differences among the models may vary. Sixth, the observed bias in this study could be influenced when we expand to a larger dataset acquired in different scanners and site locations by variations in scanning parameters (gantry tilt, tube voltage, reconstruction kernel, and slice orientation) and intravenous contrast application (time interval) (20, 52, 53). Seventh, the variations in the material strength data from Hounsfield value due to partial volume effect is not considered in the current study.

In conclusion, we have demonstrated the feasibility of using routine clinical MDCT scans to generate finite element (FE) models for the opportunistic assessment of osteoporotic fracture risk based on FE-predicted vertebral strength. We found stronger correlations between the FE-predicted bone strength measures derived from the images with different slice thicknesses and with and without intravenous contrast medium with some bias. Thus, routine clinical MDCT scans and retrospective scan data could be exploited for opportunistic screening for patients with increased risk for osteoporotic fracture using finite element analysis.

## Data Availability Statement

The raw data supporting the conclusions of this article will be made available by the authors, without undue reservation.

## Ethics Statement

The studies involving human participants were reviewed and approved by Technische Universität München institute review board. The ethics committee waived the requirement of written informed consent for participation.

## Author Contributions

NR, MD, ML, PN, JK, TB, and SK contributed conception and design of the study; SK and TB supervised the work; NR performed the finite element analysis; SK and NR performed statistical analysis; SK and NR wrote the first draft of the manuscript. All authors contributed to manuscript revision, read and approved the submitted version. All authors contributed to the article and approved the submitted version.

## Funding

This work was supported by grants of the German Research Foundation Project 32290010 (to JK, TB, and PN) and SGP Healthcare Fund, PIE-SGP-HC-2019-01 (Thrust 3-2) (to KS).

## Conflict of Interest

The authors declare that the research was conducted in the absence of any commercial or financial relationships that could be construed as a potential conflict of interest.
